# Temporal Patterns of Medications Dispensed to Children and Adolescents in a National Insured Population

**DOI:** 10.1371/journal.pone.0040991

**Published:** 2012-07-19

**Authors:** Karen L. Olson, Kenneth D. Mandl

**Affiliations:** 1 Children’s Hospital Informatics Program at Harvard-MIT Health Sciences and Technology, Division of Emergency Medicine, Children’s Hospital Boston, Boston, Massachusetts, United States of America; 2 Department of Pediatrics, Harvard Medical School, Boston, Massachusetts, United States of America; The University of Queensland, Australia

## Abstract

This study aimed to comprehensively describe prevalence and temporal dispensing patterns for medications prescribed to children and adolescents in the United States. Participants were 1.6 million children (49% female) under 18 years old enrolled in a nation-wide, employer-provided insurance plan. All medication claims from 1999–2006 were reviewed retrospectively. Drugs were assigned to 16 broad therapeutic categories. Effects of trend over time, seasonality, age and gender on overall and within category prevalence were examined. Results: Mean monthly prevalence for dispensed medications was 23.5% (range 19.4–27.5), with highest rates in winter and lowest in July. The age group with the highest prevalence was one-year-old children. On average each month, 17.1% of all children were dispensed a single drug and 6.4% were dispensed two or more. Over time, prevalence for two or more drugs did not change, but the proportion of children dispensed a single drug decreased (slope -.02%, p = .001). Overall, boys had higher monthly rates than girls (average difference 0.9%, p = .002). However, differences by gender were greatest during middle childhood, especially for respiratory and central nervous system agents. Contraceptives accounted for a large proportion of dispensed medication to older teenage girls. Rates for the drugs with the highest prevalence in this study were moderately correlated (average Pearson r.66) with those from a previously published national survey. Conclusion: On average, nearly one quarter of a population of insured children in the United States was dispensed medication each month. This rate decreased somewhat over time, primarily because proportionally fewer children were dispensed a single medication. The rate for two or more drugs dispensed simultaneously remained steady.

## Introduction

Pediatric medication use in the United States (US) is understudied, in part due to a lack of resources to address this topic. Knowledge of population parameters regarding which medications are used by specific subpopulations could guide policy decisions and the direction of further research.[1] One barrier to establishing population values is the lack of a universal country-wide system to track medication use. Large insurance claims databases can fill this void, recognizing their limitations in terms of total population covered.

We sought to comprehensively describe prevalence and temporal dispensing patterns for medications prescribed to children and adolescents in the US, focusing on the number and kinds of drugs dispensed in an insured population. We studied medication claims over an eight-year period to provide data regarding seasonality and trends over time as well as population statistics by age and gender.

## Methods

### Ethics Statement

The Committee on Clinical Investigation, which is the institutional review board at Children’s Hospital Boston, approved this study. Consent from individual participants or their guardians was not obtained. This was a retrospective study of existing data and the identities of the participants were unknown to the investigators. A waiver of consent was obtained from the review board.

### Data Source

The study population was 1,604,580 children under age 18 (49% female) enrolled in a nation-wide employer-provided health insurance plan between January 1, 1999 and December 31, 2006. The study began on April 1, 1999 to allow a three-month washout period. The number of children enrolled each month ranged from 686,521 to 985,909 (Mean 828,447). Forty-three percent of all participants were enrolled on the first study date and 21% were born afterwards. Demographic data included gender, date of birth, and zip code. Children lived across the US and most (92%) lived in the same state during their time enrolled. However, 23% of all children changed zip codes at least once. Location was not further analyzed because dates were not associated with changes. Average enrollment length was 48 months (SD 31), 94% of the children were enrolled for at least 6 months, and 17% for all 93 months. Enrollment after age 18 was not included and gaps in enrollment were possible. A total of 24,319,668 medication claims were analyzed.

### Drugs and Drug Categories

Drugs were defined as drug products, regardless of number of active ingredients. Each claim had a National Drug Code used to get the generic drug name from the VantageRx® database (Cerner Multum, Inc.), which was then used to assign each drug to one of its 19 major therapeutic categories. Three categories were dropped because there were few claims: radiologic agents (288 claims), growth hormone reserve test (10 claims), and plasma expanders (1 claim). VantageRx assigns antidepressants and antipsychotics to the category labeled psychotherapeutic agents, while stimulants such as methylphenidate are put into the category labeled central nervous system (CNS) agents. Children were dispensed 1,466 different drugs and most (93%) fell into a single therapeutic category. For drugs with multiple categories, a preferred category for this study was chosen after consultation with a pediatrician and a pediatric pharmacist.

### Measures

Data analyzed for each child consisted of one record for every date that the child was enrolled in the insurance plan with variables for the total number and generic names of drugs dispensed (if any) on each date. Filled prescriptions began on the date dispensed and continued during the followings days up to the total days supplied. If dates for the same drug overlapped, the span of the overlapping prescriptions did not exceed the last day supplied for the last prescription. Gaps between prescriptions for the same drug were left as gaps. Thus, if one calculated a medication possession ratio for a single drug (number of days dispensed divided by total days between the first and last dispensation dates), the ratio could not exceed 1.0 but could be less. A dispensing episode was defined as a series of consecutive days a drug was dispensed with no gaps. Overlapping days were only counted once. A new episode began after a gap of any length.

Prevalence was defined as the percent children dispensed medication during a time period (year, month, and week). Different denominators were used including: all enrolled children, all enrolled boys, all enrolled girls, all children in each age group, and all children in each age group by gender. Age groups were constructed by taking the integer of the child’s age. For numerator values, children were counted once during a time period if they were dispensed medication, regardless of number of days dispensed. For example, a child with a 10-day prescription that began and ended in the same month was counted once for that month. If the prescription duration spanned two months, the child was counted once in each month. This counting strategy provided a month-by-month snapshot of dispensed medication prevalence. We focused on monthly prevalence to allow study of seasonal patterns. However, some yearly statistics are presented to allow comparison with other studies.[2] Also, weekly statistics were calculated for some drugs to allow comparison with a study of actual medication use during a one-week period.[3].

### Analyses

Descriptive statistics (means) were calculated for rates by gender and age, and over time. T-tests were used to compare rates by gender. Linear regression was used to assess overall change in rates over time. Correlations were used to evaluate relations between time series by gender. Correlations were also used to compare rates in this study with rates in a published study.[3] We also provide an illustrative example of the application of times series methods with an interrupted time series analysis examining the impact of Food and Drug Administration (FDA) advisories regarding antidepressant medications (see Analysis S1 and Table S2). All analyses were performed using SAS version 9.3 (SAS Institute Inc., Cary, NC).

## Results

Twenty-four percent of the children had no medication claims. They were enrolled for shorter lengths of time (Mean 28 months, SD 26) than children with claims (Mean 55 months, SD 29). These enrollment lengths do not include possible enrollment time after age 18. Those with claims had an average of 20 claims (SD 30, max 1323) and on average, filled prescriptions during 29% of their months enrolled (SD 25).

**Figure 1:**
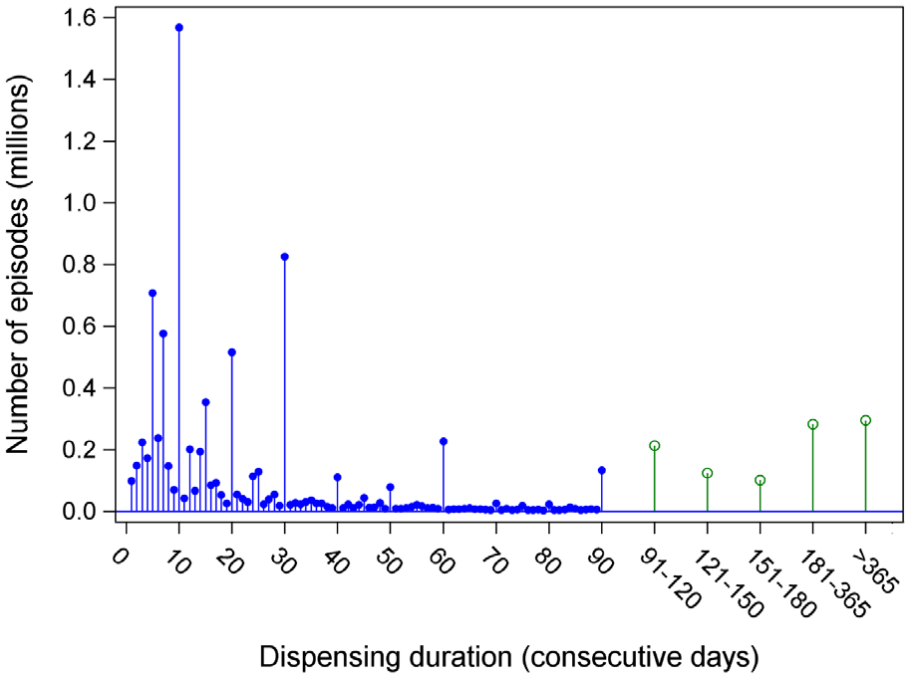
Number of consecutive days that drugs were dispensed. A dispensing episode was defined as a series of consecutive days filled. Gaps were not allowed between the end of one prescription and the beginning of another. If days overlapped, they were counted once.

Forty-eight percent of all children were dispensed medications for durations that were never greater than 90 consecutive days. Seventeen percent of all children were dispensed at least one drug for 91-365 days, and 11% for over 1 year. The duration of dispensing episodes is illustrated in Figure 1. Durations ranged from 1 to 2925 days. Most episodes (89%) were of short duration, 90 days or less. However, 3.2% of the episodes continued for over 1 year. The most frequently occurring length was 10 days (17% of all episodes), followed by 30 days (9% of all episodes).

Each year, more than half of all children had at least one filled prescription. Percents by gender and year are listed in Table 1. Girls are listed twice, once for any drug and once for any except contraceptives. T-tests comparing yearly rates by gender were not statistically significant, regardless of whether contraceptives were included or not.

**Table 1:** Percent of All Girls or All Boys Dispensed Any Drug at Any Time each Year.

Year	% Girls	% Girls *	% Boys
1999 †	52.0	51.5	51.6
2000	58.8	58.4	58.0
2001	59.7	59.1	58.9
2002	58.7	58.1	58.0
2003	60.3	59.6	59.5
2004	56.0	55.3	55.1
2005	56.6	55.9	55.8
2006	55.3	54.6	54.4

* Any drugs except contraceptives.

† 9 months, April to December.

The percent children dispensed medications in each category are presented in Table 2 in descending order. Anti-infectives and respiratory agents were most prevalent. On average, 9.4% and 8.2% of all children, respectively, were dispensed medications from these two categories each month. The twenty-five most prevalent medications are presented in Table 3. The list of drugs includes 9 anti-infectives, 8 respiratory agents, 2 CNS agents, 2 nutritional products, 2 hormones, 1 topical and 1 psychotherapeutic agent. The top five medications in each therapeutic category are presented in Table S1.

**Table 2:** Percent of All Children Dispensed Medications Each Month by Therapeutic Category.

Drug Category	% Mean	Std Dev	Min	Max
Anti-infectives	9.40	2.15	5.86	14.60
Respiratory agents	8.23	1.04	5.73	10.09
Topical agents	5.06	0.28	4.55	5.59
Central nervous system agents	4.18	0.42	2.96	4.93
Hormones	1.99	0.18	1.53	2.35
Nutritional products	1.86	0.27	1.38	2.27
Psychotherapeutic agents	1.50	0.15	1.15	1.77
Gastrointestinal agents	0.83	0.20	0.49	1.18
Cardiovascular agents	0.43	0.01	0.39	0.45
Metabolic agents	0.20	0.02	0.16	0.23
Antineoplastics	0.18	0.03	0.13	0.26
Miscellaneous agents	0.09	0.01	0.07	0.10
Immunologic agents	0.03	0.01	0.02	0.06
Alternative medicines, nutraceutical	0.01	0.00	0.01	0.01
Coagulation modifiers	0.01	0.00	0.01	0.01
Biologicals	0.00	0.00	0.00	0.01

Drugs were assigned to categories using the VantageRx® database (Cerner Multum, Inc.). Statistics for monthly prevalence (percent children dispensed medications in each category) were calculated over 93 months of study.

**Table 3:** Top 25 Medications Dispensed to Children in order of Monthly Prevalence.

Drug Name	Therapeutic Category	% Mean	Std Dev	Min	Max
Amoxicillin	Anti-infective	2.98	0.89	1.57	5.23
Cetirizine	Respiratory	1.72	0.50	0.73	2.74
Albuterol	Respiratory	1.65	0.30	1.02	2.31
Azithromycin	Anti-infective	1.49	0.53	0.67	2.76
Methylphenidate	CNS agents	1.30	0.18	0.69	1.56
Amoxicillin-clavulanate	Anti-infective	1.29	0.38	0.70	2.19
Montelukast	Respiratory	1.21	0.53	.029	2.13
Loratadine	Respiratory	0.99	1.05	0.00	3.10
Multivitamin with fluoride	Nutritional	0.90	0.14	0.64	1.11
Amphetamine-dextroamphetamine	CNS agents	0.87	0.15	0.42	1.09
Fluoride	Nutritional	0.70	0.08	0.55	0.82
Minocycline	Anti-infective	0.52	0.06	0.40	0.62
Cephalexin	Anti-infective	0.52	0.04	0.43	0.59
Fexofenadine	Respiratory	0.49	.018	0.13	0.84
Sulfamethoxazole-trimethoprim	Anti-infective	0.43	0.07	0.33	0.67
Cefprozil	Anti-infective	0.42	0.18	0.13	0.90
Cefdinir	Anti-infective	0.39	0.29	0.03	1.13
Mometasone nasal *	Topical	0.39	0.10	0.16	0.68
Fluticasone nasal	Respiratory	0.37	0.06	0.26	0.57
Prednisolone	Hormones	0.36	0.09	0.19	0.59
Ethinyl estradiol-norgestimate	Hormones	0.36	0.05	0.23	0.45
Fluticasone-salmeterol	Respiratory	0.34	0.24	0.00	0.64
Doxycycline	Anti-infective	0.33	0.05	0.20	0.40
Sertraline	Psychotherapeutic	0.32	0.05	0.23	0.44
Fluticasone	Respiratory	0.32	0.06	0.17	0.49

Abbreviation: CNS, central nervous system.

* Although mometasone was categorized as both a respiratory and a topical agent (respiratory was chosen for this paper) in the VantageRx® database (Cerner Multum, Inc.), mometasone nasal was categorized only as a topical agent.

Statistics for monthly prevalence (percent children dispensed each medication) were calculated over 93 months of study.

The top ten drugs dispensed to children in each of five age groups are presented in Tables 4 and 5. Also included in Table 4 are the top prescription drugs used by participants in a published survey of recent medication use.[3] Weekly rates are presented in these tables to allow comparison with weekly rates in the survey. If a drug from the survey was not amongst the top ten from this study, the rank order from this study is listed in Table 4. Rates for children under 12 years old dispensed each drug in the two studies were moderately correlated (average Pearson r.66, all p values <.05).

**Table 4:** Prevalence of Dispensed Medications by Age Group for Children under 12 Years Old.

	Rank order			Weekly percent	
Age	Claims	Slone*	Drug name	Claims	Slone*
0–1 y	1	1	Amoxicillin	2.46	5.1
	2	3	Multivitamin with fluoride	1.53	2.3
	3	8	Amoxicillin-clavulanate	1.22	0.7
	4	2	Albuterol	1.03	4.3
	5	9	Fluoride	0.99	0.5
	6	4	Ranitidine	0.93	1.4
	7	7	Cetirizine	0.69	0.9
	8	.	Azithromycin	0.59	.
	9	.	Cefdinir	0.54	.
	10	.	Cefprozil	0.45	.
	20	5	Hydrocortisone topical	0.24	1.3
	28	6	Pimecrolimus topical	0.15	1.0
2–5 y	1	4	Multivitamin with fluoride	1.67	2.7
	2	3	Cetirizine	1.66	2.8
	3	2	Amoxicillin	1.45	2.9
	4	7	Fluoride	1.30	1.3
	5	5	Montelukast	1.16	2.5
	6	1	Albuterol	0.75	3.4
	7	11	Amoxicillin-clavulanate	0.73	0.5
	8	8	Loratadine	0.51	0.8
	9	9	Azithromycin	0.45	0.6
	10	10	Budesonide	0.36	0.5
	15	6	Fluticasone	0.25	1.3
6–11 y	1	7	Cetirizine	1.40	1.5
	2	3	Methylphenidate	1.34	2.0
	3	4	Montelukast	1.20	1.8
	4	2	Loratadine	0.99	2.8
	5	6	Amoxicillin	0.88	1.5
	6	9	Amphetamine-dextroamphetamine	0.84	1.1
	7	5	Multivitamin with fluoride	0.79	1.8
	8	1	Albuterol	0.75	3.2
	9	.	Fluoride	0.66	.
	10	14	Amoxicillin-clavulanate	0.37	0.5
	12	10	Fluticasone-salmeterol	0.29	0.8
	13	11	Azithromycin	0.28	0.6
	14	15	Atomoxetine	0.28	0.5
	15	12	Fexofenadine	0.28	0.6
	17	8	Fluticasone	0.25	1.3

* Slone survey.[3].

Drugs listed include the ten most prevalent in the claims data as well as any other prescription drugs listed in the results from the Slone survey of children under 12 years old. For claims data, percents were averaged over the weeks of the study. Average numbers of children enrolled each week in the claims data by age group were: 0–1 year: 56,616; 2–5 years: 140,611; 6–11 years 276,085. For survey data, percents are those reporting use of each drug during the past week. Numbers of children in the Slone Survey by age group were: 0–1 year: 478; 2–5 years: 1,000; 6–11 years: 1,379. Pearson correlations of claims and survey percents were.68 (0–1 and 2–5 years) and.64 (6–11 years), p<.05 at each age.

**Table 5:** Prevalence of Dispensed Medications by Age Group for Children 12 to 17 Years Old.

Age	Rank	Drug name	Weekly percent
12–15 y	1	Methylphenidate	1.30
	2	Amphetamine-dextroamphetamine	0.86
	3	Cetirizine	0.84
	4	Albuterol	0.78
	5	Montelukast	0.70
	6	Minocycline	0.60
	7	Loratadine	0.58
	8	Amoxicillin	0.51
	9	Fexofenadine	0.50
	10	Sertraline	0.39
16–17 y	1	Ethinyl estradiol-norgestimate	1.61
	2	Minocycline	1.29
	3	Methylphenidate	0.75
	4	Doxycycline	0.70
	5	Albuterol	0.69
	6	Cetirizine	0.62
	7	Amphetamine-dextroamphetamine	0.61
	8	Sertraline	0.56
	9	Fexofenadine	0.55
	10	Amoxicillin	0.53

Drugs listed include the ten most prevalent in the claims data by age group. Percents were averaged over the weeks of the study. Average numbers of children enrolled each week were: age 12–15 years: 226,093; 16–17 years: 122,708.

**Figure 2:**
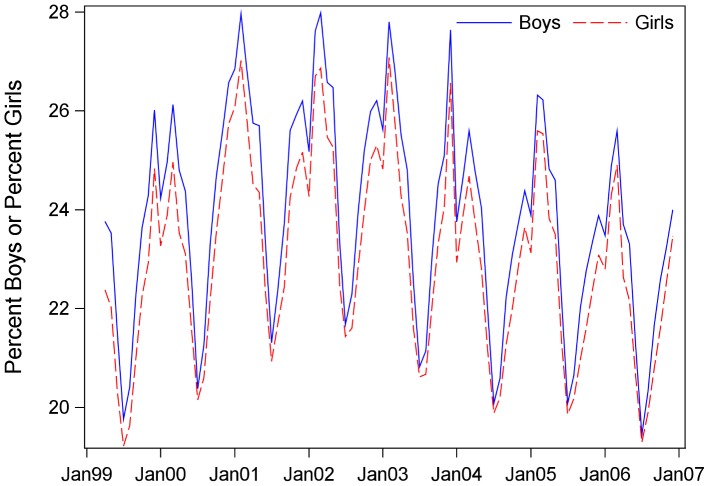
Percent of all girls and percent of all boys dispensed medication each month. Study dates were 4/1/1999 to 12/31/2006.

On average each month, 23.5% of all children were dispensed medication (SD 2.0, range 19.4 to 27.5). This is an average of 194,270 children per month (SD 25,725). Percentages were increasing in the earlier years of study and then leveled off and decreased so that rates in the final year (2006 monthly Mean 22.2%) were much like the first year (1999 monthly Mean 22.4%). Monthly rates by gender for dispensed medications are shown in Figure 2. Seasonality is apparent; peaks occurred in winter months (December to March) and July had the lowest percents each year. Boys and girls had similar seasonal patterns. Overall, a higher percent of boys (Mean 23.9%) filled prescriptions than girls (Mean 23.0%, t value −3.05, p = .002).

Children were usually dispensed one drug at a time. Averaging across the months of study, 17.1% of all children were dispensed a single drug (range 14–19.9%), 6% were dispensed two or three drugs at the same time (range 4.4–7.4%), and 0.4% four or more (range 0.2–0.5%). Among children with filled prescriptions each month, on average, 73% were dispensed one drug, 25% were dispensed two or three, and 2% four or more on the same day.

To assess whether there was a change over time for rates of polypharmacy, linear regressions were fit to monthly time series for the percents of children dispensed a single drug or more than one drug. Over the duration of study, the percent children dispensed a single drug decreased (slope −0.017, p = .001). The percent children dispensed two or more drugs did not change significantly. Therefore, the decline in the proportion of children with single-drug prescriptions was not accompanied by an increase in polypharmcy. Instead, a smaller proportion of children were dispensed medication towards the end of the study.

**Figure 3:**
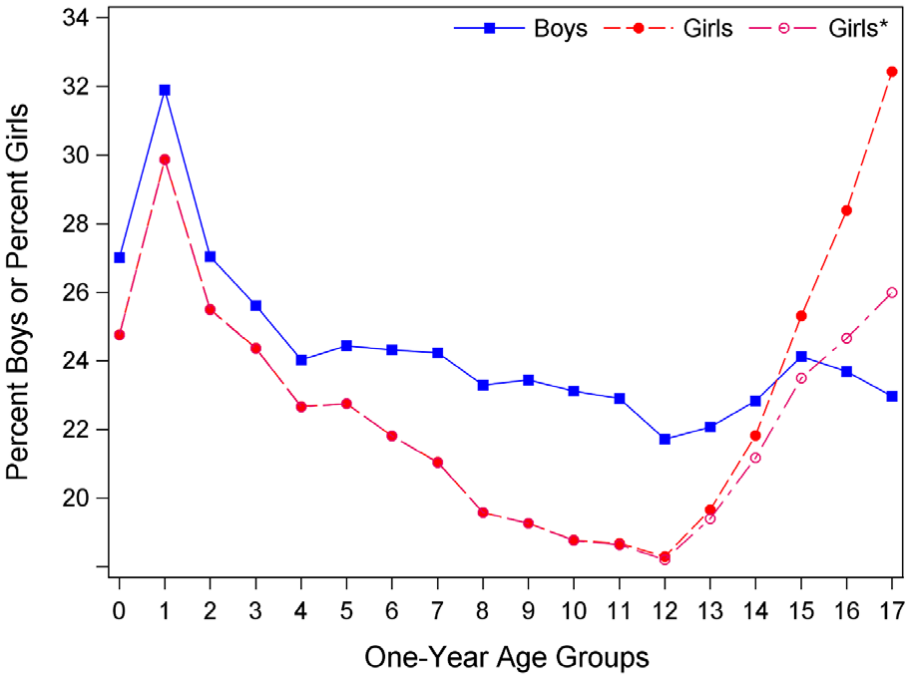
Average percent of all boys and all girls dispensed medication by one-year age groups. * Contraceptives were excluded from the list of drugs. Percents by age groups are averages over the 93 months of study.

The impact of gender and age on dispensation rates is shown in Figure 3 where percents for the 93 months of the study were averaged. Except for 17-year-old girls (32.7%), 1-year-olds had the highest percents dispensed medication (boys 31.6%, girls 29.6%). Note that when contraceptives were removed from the list of drugs, the percent of teenage girls with filled prescriptions decreased (26.3% at age 17). More boys than girls had filled prescriptions up to age 15. Percents dispensed medication declined after age 1 for both genders, but the decrease was smaller for boys, especially after age 4, up to age 12–13 when rates began to increase.

**Figure 4:**
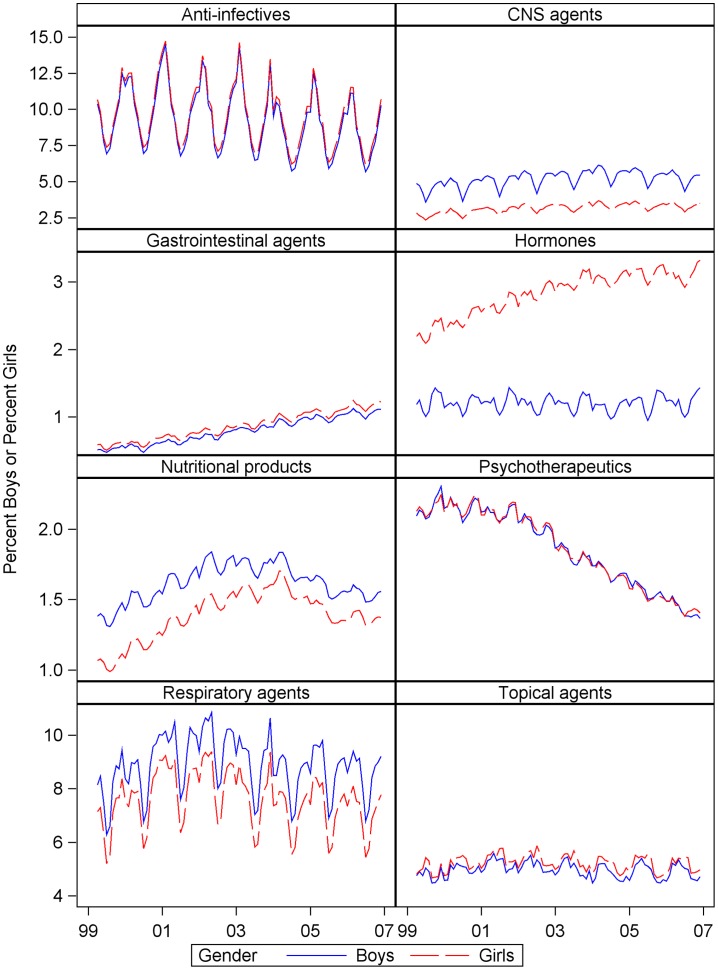
Percent of girls and boys dispensed medications in eight therapeutic categories. Study dates were 4/1/1999 to 12/31/2006.

To examine the type of drugs dispensed, the eight categories with the highest prevalence were examined (Figure 4). Monthly time series for boys and girls of CNS, respiratory, psychotherapeutic, gastrointestinal, and topical agents were highly correlated (Pearson r > = .93, p<.001) and moderately correlated for hormones (Pearson r.24, p = .02). Values for boys were greater for CNS agents (Mean difference 2%), respiratory agents (Mean difference 1.3%), and psychotherapeutic agents (Mean difference 0.2%). Values were greater for girls for hormones (Mean difference 1.6%), gastrointestinal agents (Mean difference 0.1%), and topical agents (Mean difference 0.2%). T-tests comparing gender for the six categories above were all significant (p<.001 for all except gastrointestinal where p = .01). The effect of gender on prevalence of anti-infectives and nutritional products was not statistically significant.

**Figure 5:**
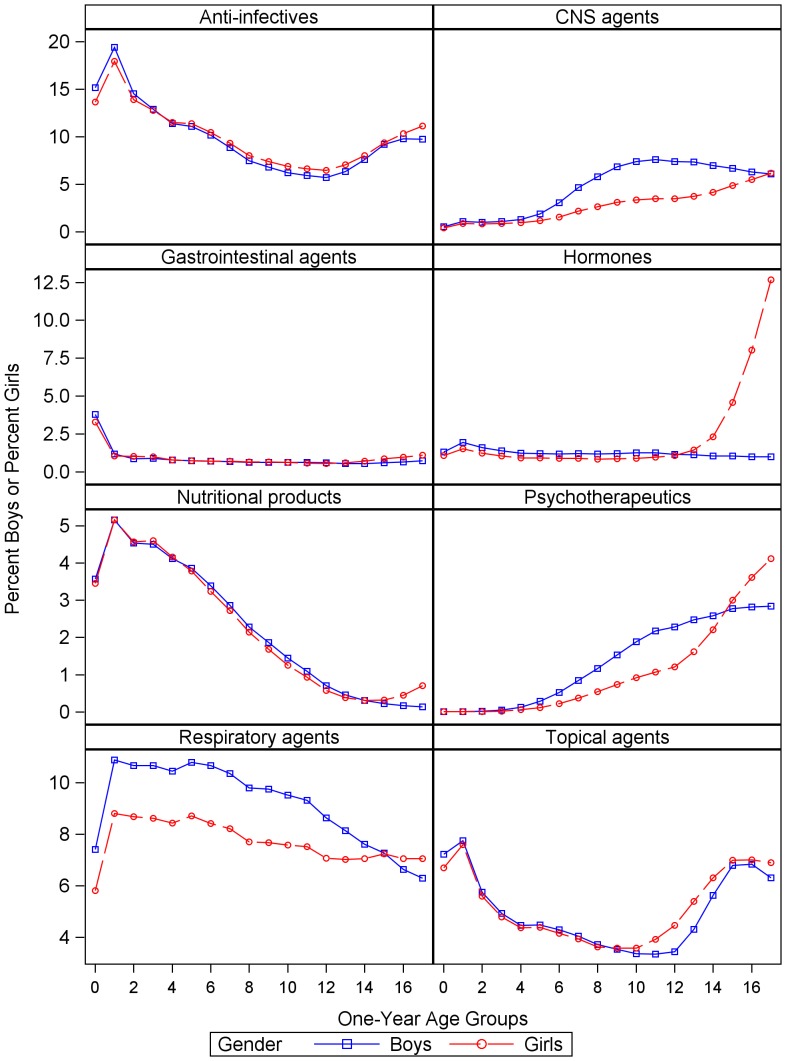
Average percents of children dispensed medications by therapeutic category, gender and one-year age groups. Percents by age groups are averages over the 93 months of study.

Age and gender effects for these eight drug categories were also examined (Figure 5). A large percent of infants (age 0–1) were dispensed anti-infectives (Mean across gender and age 16.3%), respiratory agents (Mean 8.2%), and topical agents (Mean 7.2%). The percents for anti-infectives and topical agents decreased after age 1 up to age 10–12 when they then increased over the remaining ages. Respiratory agents continued to decline or level off after infancy. Rates for gastrointestinal agents were highest for infants less than 1 year old (Mean 3.5%). They decreased sharply at age 1 and remained stable (Mean 0.7%) up to age 14.

Although a greater percent of boys than girls were dispensed CNS agents at all ages except 17 (Mean for age 0–16 for boys 4.5%, girls 2.6%), the percents by gender began to diverge after age 4 where the rate of increase for boys was greater. The largest difference by gender occurred between ages 10–12 (Mean for boys 7.4%, girls 3.4%). During the teenage years, percents for boys decreased while percents for girls continued to increase. Thus, by age 17, the difference by gender was only 0.1% and girls had the higher rate (6.2%).

Few children age 0–3 were dispensed psychotherapeutic agents (Mean across age and gender 0.02%). This category consisted of antidepressant and antipsychotic medications. From age 4 to 11, psychotherapeutic prescriptions for both genders increased, but at a higher rate for boys. The largest difference was at ages 11–12 (Mean for boys 2.2%, girls 1.2%). After age 12, the rates increased more for girls so that girls were dispensed more psychotherapeutic agents beginning at age 15. At age 17, the gender difference was 1.3% (rate for girls 4.1%).

For nutritional products, the average difference by gender was very small (0.1%) for ages 0–14 and then a somewhat greater percent of girls was dispensed these products. The average rates for hormones between ages 0–13 were 1.0% for girls and 1.3% for boys. At age 14, the percent girls dispensed hormones began to increase greatly until on average, across months, 12.7% of 17-year-old girls filled prescriptions (boys age 14–17 Mean 1.0%; girls age 14∶2.4%, age 15∶4.7%, age 16∶8.4%). This category includes contraceptives.

## Discussion

On average, almost one quarter of a population of insured children was dispensed medication each month, with highest rates in winter and lowest in July. Overall, boys had higher monthly rates, but differences by gender were greatest during middle childhood, particularly for CNS and respiratory agents. Contraceptive use contributed greatly to dispensing rates for older teenage girls. One-year-olds had notably high rates, particularly for anti-infectives, respiratory and topical agents, and nutritional products.

Few studies of pediatric prescription drug use are able to comprehensively describe usage in the US. This study uses a nation-wide sample of children covered by employer-provided insurance to comprehensively characterize dispensing patterns over an eight-year period. The inclusion of all children under 18 years and all drugs dispensed distinguishes it from other recent studies that focus on specific medication categories and age ranges.[4]–[18].

A review of studies that did include all drugs was able to include only one US study. They found yearly prevalence rates between 50.6 and 70.4%.[2] We found that half of all children were dispensed drugs each year. However, on average, about one quarter filled prescriptions each month. The proportion of children dispensed a single drug decreased over the duration of study, but there was no statistically significant change in the proportion dispensed multiple drugs. The monthly statistics of this study highlight seasonal variability, with winter peaks and lowest rates in July. Trend was also apparent. Rates increased initially but then decreased until rates in 2006 were much like those in 1999. In Italy, a similar pattern was reported.[19] Yearly Italian prescription rates started at 61.5% in 2000 and increased until 2002, but by 2006, the rate had decreased to 61%.

Several patterns we identify are consistent with results reported from the Slone survey of all medications used by children under 12 years old.[3] The Slone survey was conducted by telephone between 1998 and 2007 and included 2,857 US children assessed by parental report on use of both over-the-counter (OTC) and prescription drugs. The survey found that 20% of the children used at least one prescription drug during the past week. Our monthly rate was 23.5%. Our rate for the use of more than one drug simultaneously was 6.4%, which is similar to the Slone-reported rate of 5.9%.

In the Slone survey,[3] the three most commonly used drugs were all OTC products (acetaminophen, multivitamins, ibuprofen). The most common prescription drugs were amoxicillin, albuterol, and multivitamins with fluoride. In our study, those three drugs were ranked 1, 3, and 9 in terms of average prevalence. Our study differed from the Slone survey in that it included older children and a far greater number of children. Yet when individual drugs were examined within age groups, rates in the two studies tracked each other.

One of the strengths of this study is its detailed assessment of age and gender. More boys than girls were dispensed medication, but the average difference in rates was small. However, the size of gender differences depended on age. A greater percent of boys than girls filled prescriptions up to adolescence, a pattern also observed in Norway where yearly prevalence was reported for children age 0 to 14 years.[5] A difference in our data, however, is that prevalence decreased with age at a faster rate for girls, resulting in greater differences by gender between ages 5 to 13. The impact of one group of drugs was also noteworthy where rates for older teenage girls decreased markedly when contraceptives were excluded.

Winter peaks were expected for respiratory agents and anti-infectives, many of which are used to treat illnesses with seasonal patterns. It was less clear that CNS and psychotherapeutic agents should exhibit regular winter peaks and summer troughs. However, this pattern could be associated with use of these medications during the school year.

The higher rates for boys, compared to girls, for CNS agents during middle childhood ages are interesting in light of the finding that methylphenidate and amphetamine-dextroamphetamine were among the most prevalent drugs (Table 3). Further, although clonidine and guanfacine are both categorized as cardiovascular agents, they are often dispensed to children for attention deficit hyperactivity disorder.[20]–[22] These two drugs were the most prevalent cardiovascular agents in our study. Also, boys in this same age range had higher rates for psychotherapeutic agents (antidepressants and antipsychotics) until age 15. For girls, psychotherapeutic rates increased steadily after age 5, but began a more rapid increase after age 12. From age 15 to 17, a greater percent of girls than boys had prescriptions for psychotherapeutics. Rates for CNS agents also increase during middle childhood until they are the same as rates for boys at age 17. Thus, during middle childhood, a number of drugs prescribed for social/behavior issues have higher rates in boys but rates level off or decline in the later teenage years. But rates for girls continue to increase so that they match or exceed rates for boys at the end of the adolescent years.

In Analysis S1, we describe temporal trends for four antidepressant medications (paroxetine, fluoxetine, bupropion, sertraline). In June 2003, the FDA recommended that paroxetine not be used to treat pediatric patients with major depressive disorder. In March 2004, the FDA warned that both adults and pediatric patients be closely monitored if prescribed any of ten antidepressants that included these four. We found associations between increasing concern regarding side effects of antidepressants and utilization in this pediatric population. However, the dispensing patterns of these four medications were not independent events. Thus, reduced use of one drug may induce a rise in use of a substitute. Or awareness of risks associated with specific medications may lead to reluctance to use any drugs in the same category.

### Limitations

There is a caveat regarding the extent to which we can generalize the results of our study. Children who were uninsured, covered by government insurance, or had their prescriptions paid for in cash despite insurance were not studied. Therefore, findings from these data are most applicable to US children covered by private insurance. Because some demographic variables, such as socioeconomic status and ethnicity, were not available in these claims data, it is not possible to determine representativeness of the sample by fully comparing it with the larger US population of children.

We analyzed claims for dispensed medications, which is related to, but not strictly indicative of actual administration and adherence. However, this study provides support for the notion that prescription fills are related to adherence. Population values for the most prevalent medications were moderately correlated with values obtained from the Slone survey of reported medication use.[3] Other studies that try to correlate prescription fills with usage report associations with health outcomes rather than actual use, which we do in this study.[23]–[25].

We show that claims data can provide population parameters for pediatric medication use. For frequently dispensed medications, large samples of children can be identified. Further research could be directed at appropriate use of or outcomes associated with specific medications. Or the impact of policy recommendations such as warnings for antidepressants or specifications for appropriate antibiotic use could be examined with time series methods. Even for less frequently dispensed medication, claims aggregate data for larger and more diverse samples than typical of other studies such as clinical trials, for example. Therefore, although claims data do have limitations, they are an important resource for both policy and research efforts.

Another limitation relates to the precision of measurement. Prescriptions may have been filled prior to the first enrollment date. Therefore, the first three months of available data were not analyzed to allow time for new prescriptions to extend past the first study date. However, children could enroll any time after the first study date and their prescription histories were unknown. Thus, the number of children with filled prescriptions (numerator values) could be underestimated. This was likely most problematic in January when a large number of new enrollees were added to insurance plans.

New enrollees in January may also have contributed to an overestimate of denominator values because the insurance companies may not have yet purged their databases of children no longer enrolled. It was not unusual to observe a bump in enrollment in January with a “correction” (decrease) in February. Therefore, rates of dispensed medication to children in January may be underestimated because both the numerators were too small and the denominators too big. This contributes to the difficulty of pinpointing the exact timing of winter peaks each year. Nonetheless, a pattern of winter peaks was readily apparent as illustrated in Figure 2.

### Conclusions

On average, nearly one quarter of a population of insured children under age 18 in the US was dispensed medication in any given month. This rate decreased somewhat over time, primarily because proportionally fewer children were dispensed a single medication. The rate for two or more drugs dispensed simultaneously remained steady. Insurance claims appear to be a valuable source of population level medication data.

## Supporting Information

Table S1 **Top Five Drugs Dispensed to Children in Each Therapeutic Category.**
(DOCX)

Table S2 **Parameter Estimates for Time Series Models for Four Antidepressant Medications (Paroxetine, Fluoxetine, Sertraline, Buproprion).**
(DOCX)

Analysis S1 **Effect of Food and Drug Administration Advisories on Dispensing Patterns of Four Antidepressants Prescribed to Children.**
(DOCX)
