# Supplementary material for: Temporal Patterns of Medications Dispensed to Children and Adolescents in a National Insured Population
Source: PLoS One. 2012 Jul 19;7(7):e40991. doi: 10.1371/journal.pone.0040991 (PMC3400586; doi:10.1371/journal.pone.0040991)
Supplement: Table S1 — Top Five Drugs Dispensed to Children in Each Therapeutic Category. (DOCX) [file pone.0040991.s001.docx]

**Table S1. Top Five Drugs Dispensed to Children in Each Therapeutic Category**

|  |  | **Percent children dispensed medication** | | | |
| --- | --- | --- | --- | --- | --- |
| **Drug Category** | **Drug Name** | **% Mean** | **Std Dev** | **Min** | **Max** |
| Anti-infectives | Amoxicillin | 2.980 | 0.893 | 1.568 | 5.230 |
| (170 drugs) | Azithromycin | 1.487 | 0.533 | 0.666 | 2.763 |
|  | Amoxicillin-clavulanate | 1.291 | 0.377 | 0.699 | 2.193 |
|  | Minocycline | 0.523 | 0.063 | 0.405 | 0.625 |
|  | Cephalexin | 0.515 | 0.035 | 0.433 | 0.590 |
| Respiratory agents | Cetirizine | 1.722 | 0.498 | 0.735 | 2.738 |
| (192 drugs) | Albuterol | 1.653 | 0.301 | 1.021 | 2.314 |
|  | Montelukast | 1.214 | 0.530 | 0.295 | 2.126 |
|  | Loratadine | 0.995 | 1.053 | 0.000 | 3.103 |
|  | Fexofenadine | 0.486 | 0.178 | 0.134 | 0.836 |
| Topical agents | Mometasone nasal | 0.388 | 0.101 | 0.158 | 0.679 |
| (264 drugs) | Adapalene topical | 0.310 | 0.050 | 0.233 | 0.402 |
|  | Clindamycin topical | 0.290 | 0.056 | 0.216 | 0.393 |
|  | Benzoyl peroxide- clindamycin topical | 0.289 | 0.192 | 0.000 | 0.494 |
|  | Benzoyl peroxide topical | 0.268 | 0.040 | 0.201 | 0.354 |
| CNS agents | Methylphenidate | 1.296 | 0.180 | 0.692 | 1.556 |
| (196 drugs) | Amphetamine- dextroamphetamine | 0.872 | 0.151 | 0.417 | 1.094 |
|  | Acetaminophen-codeine | 0.267 | 0.023 | 0.225 | 0.317 |
|  | Acetaminophen-hydrocodone | 0.256 | 0.042 | 0.165 | 0.347 |
|  | Atomoxetine | 0.255 | 0.269 | 0.000 | 0.669 |
| Hormones | Prednisolone | 0.364 | 0.094 | 0.188 | 0.589 |
| (82 drugs) | Ethinyl estradiol-norgestimate | 0.362 | 0.049 | 0.229 | 0.446 |
|  | Prednisone | 0.250 | 0.028 | 0.193 | 0.320 |
|  | Levothyroxine | 0.159 | 0.015 | 0.121 | 0.179 |
|  | Desmopressin | 0.118 | 0.011 | 0.096 | 0.145 |
| Nutritional products | Multivitamin with fluoride | 0.896 | 0.140 | 0.643 | 1.106 |
| (63 drugs) | Fluoride | 0.702 | 0.081 | 0.546 | 0.821 |
|  | Multivitamin with iron and fluoride | 0.133 | 0.046 | 0.062 | 0.209 |
|  | Sodium chloride | 0.033 | 0.015 | 0.011 | 0.069 |
|  | Multivitamin, prenatal | 0.033 | 0.006 | 0.025 | 0.046 |
| Psychotherapeutics | Sertraline | 0.321 | 0.048 | 0.228 | 0.437 |
| (46 drugs) | Fluoxetine | 0.221 | 0.016 | 0.178 | 0.245 |
|  | Bupropion | 0.178 | 0.021 | 0.139 | 0.221 |
|  | Paroxetine | 0.164 | 0.067 | 0.061 | 0.271 |
|  | Risperidone | 0.163 | 0.027 | 0.100 | 0.195 |
| Gastrointestinal agents | Ranitidine | 0.210 | 0.026 | 0.148 | 0.265 |
| (56 drugs) | Polyethylene glycol 3350 | 0.169 | 0.126 | 0.000 | 0.383 |
|  | Lansoprazole | 0.120 | 0.088 | 0.034 | 0.297 |
|  | Omeprazole | 0.053 | 0.029 | 0.016 | 0.094 |
|  | Metoclopramide | 0.052 | 0.010 | 0.027 | 0.067 |
| Cardiovascular agents | Clonidine | 0.176 | 0.007 | 0.161 | 0.194 |
| (124 drugs) | Guanfacine | 0.064 | 0.004 | 0.054 | 0.074 |
|  | Propranolol | 0.038 | 0.005 | 0.028 | 0.047 |
|  | Atenolol | 0.024 | 0.002 | 0.020 | 0.028 |
|  | Enalapril | 0.019 | 0.004 | 0.013 | 0.026 |
| Metabolic agents | Insulin lispro | 0.087 | 0.012 | 0.066 | 0.110 |
| (62 drugs) | Insulin isophane | 0.061 | 0.023 | 0.029 | 0.100 |
|  | Insulin glargine | 0.032 | 0.024 | 0.000 | 0.068 |
|  | Insulin aspart | 0.025 | 0.026 | 0.000 | 0.074 |
|  | Metformin | 0.025 | 0.011 | 0.007 | 0.040 |
| Antineoplastics | Isotretinoin | 0.133 | 0.036 | 0.074 | 0.221 |
| (48 drugs) | Methotrexate | 0.017 | 0.003 | 0.009 | 0.022 |
|  | Mercaptopurine | 0.016 | 0.003 | 0.007 | 0.020 |
|  | Leuprolide | 0.010 | 0.001 | 0.007 | 0.012 |
|  | Hydroxyurea | 0.002 | 0.001 | 0.001 | 0.003 |
| Miscellaneous agents | Oxybutynin | 0.040 | 0.003 | 0.035 | 0.047 |
| (59 drugs) | Phenazopyridine | 0.020 | 0.002 | 0.015 | 0.027 |
|  | Tolterodine | 0.009 | 0.003 | 0.004 | 0.013 |
|  | Etanercept | 0.004 | 0.002 | 0.001 | 0.008 |
|  | Bethanechol | 0.003 | 0.001 | 0.002 | 0.004 |
| Immunologic agents | Azathioprine | 0.008 | 0.001 | 0.006 | 0.010 |
| (67 drugs) | Tacrolimus | 0.007 | 0.001 | 0.005 | 0.010 |
|  | Mycophenolate mofetil | 0.006 | 0.001 | 0.004 | 0.009 |
|  | Cyclosporine | 0.005 | 0.001 | 0.004 | 0.007 |
|  | Hepatitis B vaccine | 0.002 | 0.001 | 0.000 | 0.004 |
| Alternative medicines, neutraceutical | Levocarnitine | 0.011 | 0.001 | 0.009 | 0.012 |
| (6 drugs) | Omega-3 polyunsaturated fatty acids | 0.000 | 0.000 | 0.000 | 0.001 |
|  | Betaine | 0.000 | 0.000 | 0.000 | 0.000 |
|  | Inositol | 0.000 | 0.000 | 0.000 | 0.000 |
|  | Methionine | 0.000 | 0.000 | 0.000 | 0.000 |
| Coagulation modifiers | Warfarin | 0.007 | 0.001 | 0.005 | 0.009 |
| (22 drugs) | Enoxaparin | 0.001 | 0.000 | 0.000 | 0.002 |
|  | Aminocaprioic acid | 0.001 | 0.000 | 0.000 | 0.001 |
|  | Heparin | 0.001 | 0.000 | 0.000 | 0.002 |
|  | Clopidogrel | 0.001 | 0.000 | 0.000 | 0.001 |
| Biologicals | Filgrastim | 0.001 | 0.001 | 0.001 | 0.003 |
| (9 drugs) | Epoetin alfa | 0.001 | 0.000 | 0.001 | 0.002 |
|  | Secretin | 0.000 | 0.000 | 0.000 | 0.002 |
|  | Darbepoetin alfa | 0.000 | 0.000 | 0.000 | 0.001 |
|  | Sargramostim | 0.000 | 0.000 | 0.000 | 0.001 |

Abbreviation: CNS, central nervous system.

Number of drugs in each category is listed below the category name. Statistics for monthly prevalence (percent children dispensed each medication) were calculated over 93 months of study.
