# Supplementary material for: Temporal Patterns of Medications Dispensed to Children and Adolescents in a National Insured Population
Source: PLoS One. 2012 Jul 19;7(7):e40991. doi: 10.1371/journal.pone.0040991 (PMC3400586; doi:10.1371/journal.pone.0040991)
Supplement: Table S2 — Parameter Estimates for Time Series Models for Four Antidepressant Medications (Paroxetine, Fluoxetine, Sertraline, Buproprion). (DOCX) [file pone.0040991.s002.docx]

**Table S2. Parameter Estimates for Time Series Models for Four Antidepressant Medications (Paroxetine, Fluoxetine, Sertraline, Buproprion)**

| **Parameter** | **Estimate** | **Std Error** | **p-value** |
| --- | --- | --- | --- |
| Antidepressant: Paroxetine |  |  |  |
| Intercept | 0.1359 | 0.0110 | <.001 |
| Month ^1^ | 0.0050 | 0.0010 | <.001 |
| Month-squared ^2^ | -0.0001 | 0.0000 | 0.002 |
| January | -0.0048 | 0.0016 | 0.003 |
| February | -0.0002 | 0.0021 | 0.917 |
| March | 0.0064 | 0.0024 | 0.010 |
| April | 0.0081 | 0.0026 | 0.003 |
| May | 0.0059 | 0.0027 | 0.032 |
| June | -0.0016 | 0.0027 | 0.565 |
| July | -0.0082 | 0.0027 | 0.003 |
| August | -0.0083 | 0.0025 | 0.001 |
| September | -0.0065 | 0.0023 | 0.006 |
| October | -0.0023 | 0.0020 | 0.254 |
| November | -0.0012 | 0.0015 | 0.424 |
| First intervention ^3^ | -0.0159 | 0.0048 | 0.001 |
| Month at/after intervention ^4^ | -0.0076 | 0.0022 | 0.001 |
| Month at/after intervention-squared | 0.0002 | 0.0003 | <.001 |
| First order autoregression | -0.9231 | 0.0595 | <.001 |
| Antidepressant: Fluoxetine |  |  |  |
| Intercept | 0.2349 | 0.0099 | <.001 |
| Month ^1^ | -0.0004 | 0.0002 | 0.077 |
| January | -0.0048 | 0.0013 | <.001 |
| February | 0.0000 | 0.0018 | 0.991 |
| March | 0.0082 | 0.0020 | <.001 |
| April | 0.0071 | 0.0022 | 0.002 |
| May | 0.0043 | 0.0023 | 0.064 |
| June | -0.0014 | 0.0023 | 0.556 |
| July | -0.0082 | 0.0022 | <.001 |
| August | -0.0122 | 0.0021 | <.001 |
| September | -0.0113 | 0.0019 | <.001 |
| October | -0.0051 | 0.0017 | 0.003 |
| November | -0.0020 | 0.0012 | 0.110 |
| Second intervention ^5^ | -0.0043 | 0.0039 | 0.270 |
| Month at/after second intervention ^6^ | 0.0018 | 0.0006 | 0.005 |
| First order autoregression | -.09441 | 0.0361 | <.001 |
| Antidepressant: Bupropion |  |  |  |
| Intercept | 0.1557 | 0.1118 | <.001 |
| Month ^1^ | 0.0009 | 0.0003 | 0.004 |
| January | -0.0068 | 0.0014 | <.001 |
| February | -0.0018 | 0.0019 | 0.349 |
| March | 0.0071 | 0.0022 | 0.002 |
| April | 0.0078 | 0.0023 | 0.001 |
| May | 0.0097 | 0.0024 | <.001 |
| June | 0.0005 | 0.0024 | 0.852 |
| July | -0.0137 | 0.0024 | <.001 |
| August | -0.0162 | 0.0023 | <.001 |
| September | -0.0131 | 0.0021 | <.001 |
| October | -0.0070 | 0.0018 | <.001 |
| November | -0.0023 | 0.0013 | 0.086 |
| Second intervention ^5^ | -0.0026 | 0.0042 | 0.532 |
| Month at/after second intervention ^6^ | -0.0026 | 0.0007 | <.001 |
| First order autoregression | -0.9526 | 0.0338 | <.001 |
| Antidepressant: Sertraline |  |  |  |
| Intercept | 0.2382 | 0.0057 | <.001 |
| Month ^1^ | 0.0025 | 0.0005 | <.001 |
| Month-squared ^2^ | 0.0000 | 0.0000 | 0.887 |
| January | -0.0099 | 0.0019 | <.001 |
| February | -0.0023 | 0.0025 | 0.372 |
| March | 0.0074 | 0.0028 | 0.011 |
| April | 0.0069 | 0.0031 | 0.027 |
| May | 0.0094 | 0.0031 | 0.004 |
| June | -0.0024 | 0.0031 | 0.445 |
| July | -0.0151 | 0.0031 | <.001 |
| August | -0.0180 | 0.0030 | <.001 |
| September | -0.0155 | 0.0027 | <.001 |
| October | -0.0063 | 0.0024 | 0.010 |
| November | -0.0032 | 0.0018 | 0.082 |
| First intervention ^3^ | -0.0207 | 0.0072 | 0.005 |
| Month at/after intervention ^4^ | 0.0174 | 0.0041 | <.001 |
| Month at/after intervention-squared | -0.0012 | 0.0004 | 0.002 |
| Second intervention ^5^ | -0.0080 | 0.0057 | 0.166 |
| Month at/after second intervention ^6^ | -0.0072 | 0.0036 | 0.048 |
| Month at/after second intervention-squared | 0.0014 | 0.0004 | 0.004 |
| First order autoregression | -0.7012 | 0.0930 | <.001 |

1 Linear trend, incremental month of study, begins at 1 in April 1999

2 Quadratic trend, squared month-of-study value

3 After FDA recommendation regarding paroxetine, equals 1 on and after August 2003

4 Linear trend after FDA recommendation, incremental month of study, begins at 1 in August 2003

5 After FDA warning regarding ten antidepressants, equals 1 on and after May 2004

6 Linear trend after FDA warning, incremental month of study, begins at 1 in May 2004

Seasonality is represented by eleven indicator variables set to 1 for each month from January to November.
