# Supplementary material for: Temporal Patterns of Medications Dispensed to Children and Adolescents in a National Insured Population
Source: PLoS One. 2012 Jul 19;7(7):e40991. doi: 10.1371/journal.pone.0040991 (PMC3400586; doi:10.1371/journal.pone.0040991)
Supplement: Analysis S1 — Effect of Food and Drug Administration Advisories on Dispensing Patterns of Four Antidepressants Prescribed to Children. (DOCX) [file pone.0040991.s003.docx]

**Supplementary Analysis S1. Effect of Food and Drug Administration Advisories on Dispensing Patterns of Four Antidepressants Prescribed to Children**

**Introduction.** This analysis is an illustrative example of why discerning temporal trends in pharmacy prescribing data is fundamental to pharmacoepidemologic modeling. We performed an interrupted time series analysis[1] to examine the impact of United States Food and Drug Administration (FDA) advisories regarding antidepressant medications on dispensing patterns of those medications. In June 2003, the FDA recommended that paroxetine not be used to treat pediatric patients with major depressive disorder. In March 2004, the FDA warned that both adults and pediatric patients be closely monitored if prescribed any of ten antidepressants. Using claims-based population level time series, we sought to demonstrate the association of the advisories with changes in dispensing patterns of four prevalent antidepressants.

**Method.** The medications studied were paroxetine, fluoxetine, bupropion, and sertraline. An autoregressive model was fit for each drug including terms for first and second order autoregression, indicator variables for month, terms for linear and quadratic trend, and terms for level and trend changes after July 2003 (one month after the first FDA recommendation) using data from April 1999 to February 2004 (one month before the FDA warning). Backward elimination techniques were used to remove non-significant parameters. The best model for each drug was then applied to the entire time span, adding parameters for level and trend changes after April 2004 (one month after the warning). A final test of log-likelihood differences was performed to compare models with and without the intervention parameters for the second FDA warning. Analyses were performed using SAS 9.3 (SAS Institute, Inc., Cary NC).

**Results.** The final model for paroxetine included quadratic trend, seasonal effects, first order autoregression and level and trend changes after July, but no additional changes after the second warning in March 2004 (p=.20). Before the advisory, this drug was dispensed to an increasingly greater proportion of pediatric patients over time, although that trend was beginning to level off or decline. Afterwards, the pattern reversed so that a smaller proportion of patients were dispensed paroxetine by the end of the study. Parameter estimates are presented in Table S2.

The other three drugs were not included the 2003 advisory, but were in 2004. The final model for fluoxetine included linear trend, seasonal effects, and first order autoregression (see Table S2). Although there was a linear trend after the second warning, a model that removed all terms for the second intervention did not differ significantly from one that included them (p=.10).

For bupropion, the final model included linear trend, seasonal effects, first order autoregression, and a significant change in trend after the 2004 warning. Comparison tests removing terms for the second intervention indicated that the model with better fit retained these parameters (p=.03). An increasingly greater proportion of children were dispensed this medication before the warning, but dispensing rates decreased over time afterwards. Parameter estimates are presented in Table S2.

Dispensing rates for sertraline changed after both FDA advisories. The final model for sertraline included quadratic trend, seasonal effects, first order autoregression, level and trend changes after the first FDA advisory, and trend changes after the second (see Table S2). Comparison tests removing parameters for the second intervention indicated that keeping them in the model resulted in a better fit (p<.001). Before the 2003 advisory, sertraline was dispensed to an increasingly greater proportion of children over time. This trend may have been diminishing or leveling off, but rates increased after the paroxetine recommendation. After the 2004 warning for ten drugs that included sertraline, dispensing rates decreased. This decrease continued for at least a year but slowed towards the end of the study.

**Discussion.** We demonstrated a strong association between heightened concern regarding side effects of antidepressants after two FDA advisories and the utilization of antidepressants in a pediatric population.

Prescribing patterns were undoubtedly affected by other advisories and secular trends. For example, the Committee on the Safety of Medicines in the United Kingdom also advised against the use of paroxetine for major depressive disorder in 2003 and like the FDA, continued to update its list of medications and recommendations over time.[2] Furthermore, prescribing of these four antidepressants are not independent events. Reduced use of one drug may induce a rise in use of a substitute. Or awareness of risks associated with specific medications may lead to reluctance to use any drugs in the same category.

Time series modeling of dispensed medications elucidated the impact of FDA advisories on medication use. Use of claims data to model of the impact of season and demographic factors on medication prescribing will aid study of the impact of policies, advisories, and formulary decisions.

**References.**

1. Wagner AK, Soumerai SB, Zhang F, Ross-Degnan D (2002) Segmented regression analysis of interrupted time series studies in medication use research. Journal of Clinical Pharmacy and Therapeutics 27: 299-309.

2. Wagner KD, Asarnow JR, Vitiello B, Clarke G, Keller M, et al. (2012) Out of the black box: treatment of resistant depression in adolescents and the antidepressant controversy. Journal of Child and Adolescent Psychopharmacology 22: 5-10.
